# Fetus in fetu: A report of two cases

**DOI:** 10.4103/0971-9261.42572

**Published:** 2008

**Authors:** Ibrahim Karaman, Derya Erdoğan, Semire Özalevli, Ayşe Karaman, y Hakan Çavuşoğlu, M. Kemal Aslan, Özden Çakmak

**Affiliations:** Department of Pediatric Surgery, Dr. Sami Ulus Children's Hospital, Ankara, Turkey

**Keywords:** Fetus in fetu, newborn, teratoma

## Abstract

Fetus in fetu is a rare condition that has been defined as the presence of one of the twins in the body of the other. It is most frequently located in retroperitoneal area; however, it has been reported in other locations as well. This report presents two cases of “fetus in fetu” cases: one located in the retroperitoneal area and the other in the sacrococcygeal area.

Fetus in fetu (FIF) is a rare congenital anomaly. It is a condition in which malformed and parasitic fetus is located in the body of its twin. The anomaly was first defined in early nineteenth century by Meckel.[1,2] Despite its prevalence among infants and children, there have been reports of cases in which the anomaly had remained asymptomatic until later ages.[3–5] This rare congenital anomaly, which was reported around 100 times since its first definition in the nineteenth century, has been discussed with respect to its prognosis and treatment in the light of the relevant literature.

## CASE REPORTS

### Case 1

A 10-day-old female newborn, born to a 28-year-old mother at the forty-first week through normal delivery as the fourth live birth was referred to our hospital with a prediagnosis of abdominal mass. Upon physical examination, a mass with a size of nearly 10 × 15 cm was detected at the right upper quadrant of the abdomen.

**Figure 1:**
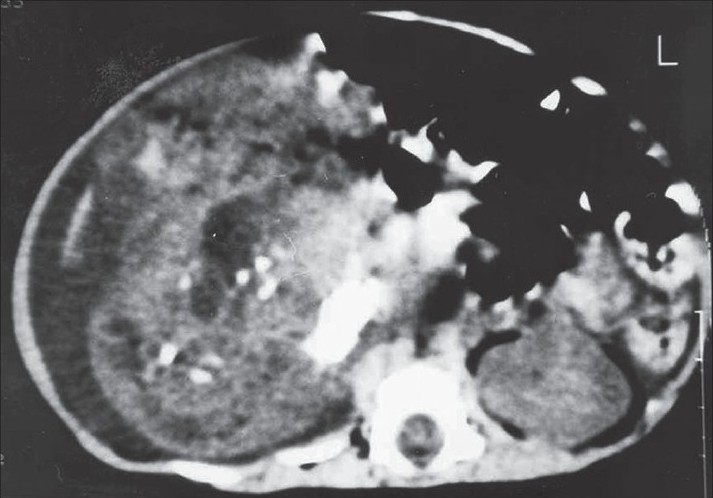
Computerized abdominal tomography shows a multilobed heterogeneous mass lesion.

The computerized abdominal tomography showed a multilobed heterogeneous mass lesion with a size of 10 × 7 × 6 cm in the retroperitoneal area [Figure 1].

The tumor markers of the patient (α-FP, CEA, NSE, β-HCG) and VMA creatinine ratio were normal.

**Figure 2:**
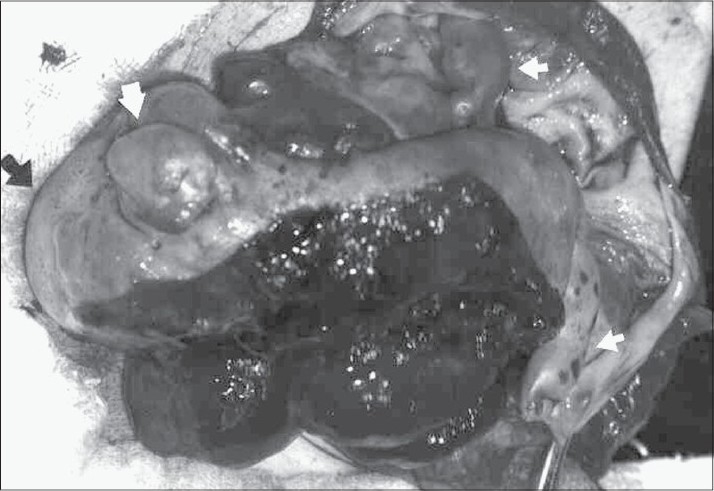
Fetiform presentation of the mass. Structures representing head of fetus (thin arrows) and two legs and an arm (bold arrows)

During the operation, a mass with a size of 10 × 15 cm was found at the retroperitoneal area. Upon the incision of the capsule, we found a fetal head, a trunk and an arm and two leg-like structures representing the extremities along with nearly 200 cc of serohemorragic fluid. The fetus, except for its ventral side, was covered by vernix caseosa [Figure 2]. The mass was totally removed, including its capsule.

Pathological studies revealed noncalcified vertebral bodies on the midline of the fetus, extending at cranial to caudal direction in the sagittal plane. The microscopic evaluation revealed skin, skin extensions, glial tissue, striated muscle, mature cartilage, peripheral nerve, lung tissue, bone and bone marrow tissues in the capsule, which were mostly lined with multifold epithelium, and at some locations, single-layered epithelium was found.

The postoperative period of the patient was free of complications. The patient who was followed-up with ultrasound and α-feto-protein estimations is currently 4 years of age and has no complaints.

### Case 2

A 3600-g, 28-day-old male term baby, born through spontaneous vaginal delivery to a mother of 29 years of age in the first pregnancy, presented to our hospital with a lump at the deeper part of the buttocks that was noticed by his mother incidentally on the third week of the birth. On physical examination, the physical appearance of the baby was normal. There was a semimobile mass of 8 × 8 cm, which could only be noticed by deep palpation. The direct radiography revealed a bony structure in the sacrum area. The abdominal ultrasound and α-FP levels were normal.

**Figure 3:**
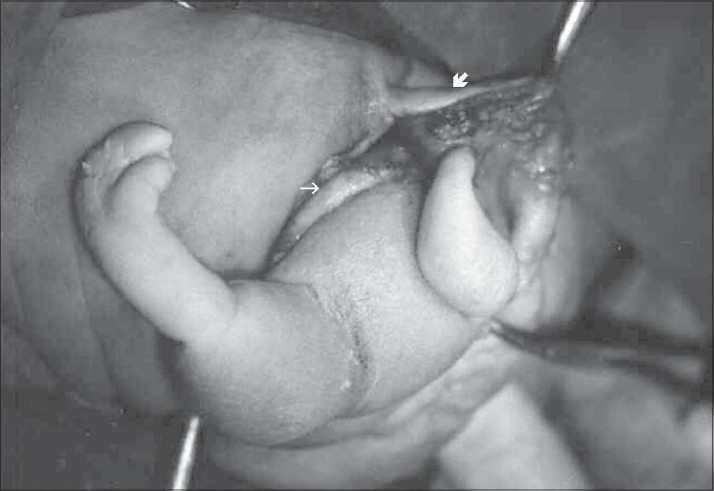
Well-developed lower extremity (bold arrow indicates the opened skin and thin arrow indicates the capsule of the mass)

During the operation, following the incision of the capsule of the mass, which presented adherence to some locations on the subcutaneous tissue, drainage of a yellowish fluid was observed. In the capsule, there was a hairy trunk with a size of 5-6 cm and two extremities, which had fingers and nails at the ends. The mass was totally removed along with the coccyx [Figure 3].

Pathological examination demonstrated skin, skin extensions, bone, bone marrow, fat tissue, venous structures, striated muscle and peripheral nerve sections. The postoperative period of the patient was uneventful. The patient is currently 5 years of age and is being periodically followed up with checkups on the α-FP levels and ultrasound and has no problems.

## DISCUSSION

Fetus in fetu malformation has been defined as the existence of a parasitic, monozygotic, diamniotic fetus in the body of its twin. Preceded by Willis in 1935, in 1954, Lord claimed the presence of a vertebral column and extremities and organs located at appropriate places around it as the basic diagnostic for FIF. These criteria are still, to a wide extent, valid today.[1,2,6–9] However, there are those who claim this pathology to be a teratoma that is well-differentiated and highly organized.[10]

Fetus in fetu most frequently (80%) inhabits the retroperitoneal region. However, there have been few reports of FIF location in the head, sacrum, scrotum and the mouth. Despite the requirement of the presence of a vertebral column for diagnosis, there are reports of the cases without a vertebral column.[7]

Fetus in fetu is considered as a benign condition. Consequently, some researchers have stated that to facilitate the excision, it is possible to leave some sections of the capsule in its place. Nevertheless, in one case, the mass has been reported to recur as a yolk sac tumor after 4 months. This has been attributed to the presence of immature tissues in the small areas and the remnants of the capsule of the mass.[1]

In summary, FIF is considered as a benign condition, while the potentially malignant characteristics of teratoma constitute the basis of the discussion. This argument may lead to differences in the follow-up and treatment of such cases. Although there is a consensus on FIF being a benign condition, considering the reports of malignant recurrences, as stated above, there is a need for the total removal of the mass including its capsule. In addition, we consider the evaluation of the postoperative tumor markers and periodical ultrasound examinations is an appropriate approach.
